# Understanding physicians’ behavior toward alerts about nephrotoxic medications in outpatients: a cross-sectional analysis

**DOI:** 10.1186/1471-2369-15-200

**Published:** 2014-12-15

**Authors:** Insook Cho, Sarah P Slight, Karen C Nanji, Diane L Seger, Nivethietha Maniam, Patricia C Dykes, David W Bates

**Affiliations:** The Center for Patient Safety Research and Practice, Division of General Internal Medicine, Brigham and Women’s Hospital, Boston, MA USA; Nursing Department, Inha University, Incheon, South Korea; Harvard Medical School, Boston, MA USA; Partners Healthcare Systems, Inc, Wellesley, MA USA; Division of Primary Care, University of Nottingham, Nottingham, UK; Department of Anesthesia, Critical Care and Pain Medicine, Massachusetts General Hospital, Boston, MA USA

**Keywords:** Medication safety, Clinical decision support system, Renal insufficiency, Drug prescribing, Chronic kidney disease

## Abstract

**Background:**

Although most outpatients are relatively healthy, many have chronic renal insufficiency, and high override rates for suggestions on renal dosing have been observed. To better understand the override of renal dosing alerts in an outpatient setting, we conducted a study to evaluate which patients were more frequently prescribed contraindicated medications, to assess providers’ responses to suggestions, and to examine the drugs involved and the reasons for overrides.

**Methods:**

We obtained data on renal alert overrides and the coded reasons for overrides cited by providers at the time of prescription from outpatient clinics and ambulatory hospital-based practices at a large academic health care center over a period of 3 years, from January 2009 to December 2011. For detailed chart review, a group of 6 trained clinicians developed the appropriateness criteria with excellent inter-rater reliability (*κ* = 0.93). We stratified providers by override frequency and then drew samples from the high- and low-frequency groups. We measured the rate of total overrides, rate of appropriate overrides, medications overridden, and the reason(s) for override.

**Results:**

A total of 4120 renal alerts were triggered by 584 prescribers in the study period, among which 78.2% (3,221) were overridden. Almost half of the alerts were triggered by 40 providers and one-third was triggered by high-frequency overriders. The appropriateness rates were fairly similar, at 28.4% and 31.6% for high- and low-frequency overriders, respectively. Metformin, glyburide, hydrochlorothiazide, and nitrofurantoin were the most common drugs overridden. Physicians’ appropriateness rates were higher than the rates for nurse practitioners (32.9% vs. 22.1%). Physicians with low frequency override rates had higher levels of appropriateness for metformin than the high frequency overriders (*P* = 0.005).

**Conclusion:**

A small number of providers accounted for a large fraction of overrides, as was the case with a small number of drugs. These data suggest that a focused intervention targeting primarily these providers and medications has the potential to improve medication safety.

## Background

Adverse drug events due to dosing errors are common, costly, and often preventable in patients with renal insufficiency [1, 2]. In primary care settings, specifically, chronic renal impairment is often discovered and diagnosed, and patients commonly have risk factors for chronic kidney disease (CKD) such as age > 65 years, hypertension, cardiovascular disease, and diabetes mellitus [3–5]. The prevalence of CKD is growing most rapidly in people aged ≥60 years. According to the kidney disease statistics in the United States, the prevalence of CKD in people aged ≥ 60 years increased from 18.8% to 24.5%, as reported in the 1988–1994 National Health and Nutrition Examination Survey (NHANES) study and the 2003–2006 NHANES study [6]. Furthermore, many patients with CKD are not appropriately diagnosed. A previous study showed that completeness of patients’ problem list in terms of renal insufficiency in one network was only 4.7%, which is lower than that for hypertension, diabetes, and breast cancer [7]. This finding suggests that their providers may not be aware of the CKD in these patients, even though the glomerular filtration rate (GFR) can be readily estimated from data routinely available in the electronic health records. Automated clinical decision support (CDS) has shown promise in reducing medication errors including improvement in the frequency of appropriate dosing [6]. However, previous studies have shown that providers override 50–80% of alerts generated by renal decision support systems. [7–9] These high override rates imply that either too many alerts are being delivered or providers may be overriding clinically important suggestions. Some providers may be especially likely to override alerts, and thus, there is scope for improvement.

We developed a renal alerting application first in inpatients with renal insufficiency and showed that making suggestions to providers improved the choices regarding dose and frequency, and reduced the length of stay among patients with renal insufficiency [8]. We subsequently applied this application to an outpatient setting. Although we have extensively limited the drug coverage to promote user acceptance, the fraction of prescriptions deemed inappropriate was high [10].

Regarding the alert overrides, many are clinically justifiable, but many are likely inappropriate. It is uncertain what the optimal override rate should be. Systems should carefully consider which warnings they choose to display. To better understand this issue, we aimed at (1) identifying which providers more frequently prescribe contraindicated medications, (2) assessing providers’ responses to renal alerts of CDS and how many overrides were considered to be appropriate, and (3) examining the drugs and reason(s) for rejecting advice provided by the renal alert system.

## Methods

A cross-sectional observational study design involving retrospective medical chart review was used in this study. This work was performed after acquiring permission from the IRB of Partners Healthcare.

### Study setting

This study included 36 primary care practices affiliated with Brigham and Women’s Hospital and Massachusetts General Hospital, which are 2 teaching hospitals of the Partners HealthCare System. A total of 1,718 prescribers serve these sites.

The Longitudinal Medical Record (LMR) is a Partners-developed electronic medical record that was implemented in 2000. The LMR has CDS capability in the form of medication alerts for drug suggestions for patients with renal insufficiency and provides 5 types of suggestions including drug-drug interaction, drug-allergy, geriatrics, duplicate therapy, and drug formulary. The LMR first determines whether a patient has renal insufficiency, which is defined as an estimated creatinine clearance (CrCL) of <50 mL/min, by the Cockroft-Gault equation. Subsequently, based on the real-time calculation of the estimated CrCL and the drug prescribed, the LMR modifies the abovementioned dose list, default dose amount, and default frequency.

**Figure 1:**
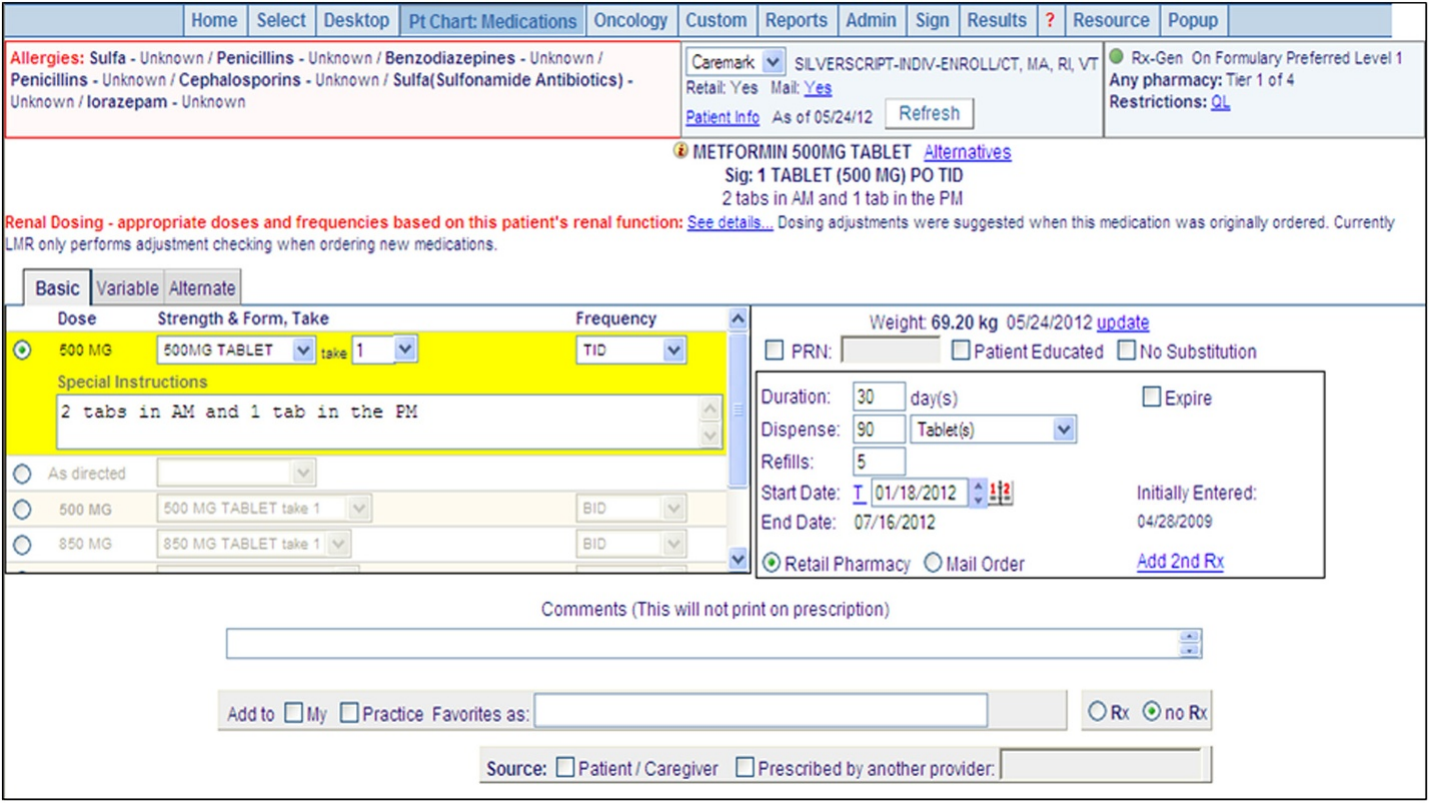
**Example of the renal-dosing CDS function of the LMR.** The “see details” link shows the value of estimated CrCL with relevant information. The “Alternatives” link located in the upper portion of the screen displays the substitute drug list.

The knowledge base was first developed approximately 15 years ago for an inpatient setting through literature reviews and an expert panel including a nephrologist, a pharmacist, and a general internist convened to review all medication in the hospital’s drug formulary; medications that were renally cleared and/or nephrotoxic were selected [8]. The knowledge base includes approximately 356 medications in 3 categories: mild (estimated GFR [eGFR], 51–80 mL/min, 26 medications), moderate (eGFR, 16–50 mL/min, 146 medications), and advanced (eGFR, ≤15 mL/min, 184 medication). For the outpatient setting, the mild category of renal insufficiency was inactivated to decrease relatively unimportant alerts and increase user adherence to alerts. The estimated CrCL was calculated from weight and age, which were reported by the nurse or physician, and the serum creatinine level as per recent laboratory measurements. If renal insufficiency was detected and any medication was ordered, the LMR modified one or more entities from among the dose list, default dose amount, and default frequency. Moreover, it showed the highlighted link introducing substitute medications to guide a user (Figure 1). For example, when a patient with estimated CrCL < 30 mL/min was prescribed hydrochlorothiazide, the “Alternatives” link shows furosemide as an alternative drug.

### Override appropriateness criteria

**Figure 2:**
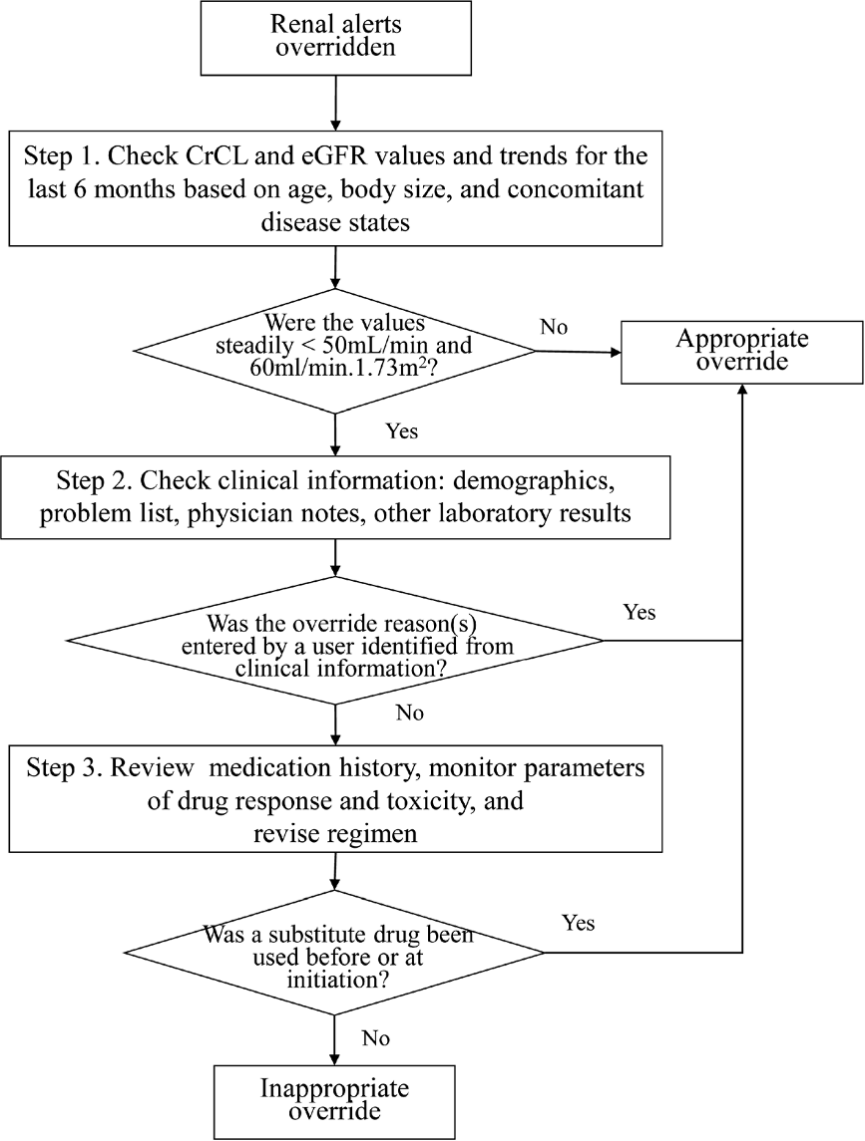
**Criteria of judgment of appropriateness for renal alert override.** CrCL: creatinine clearance.

To set up the criteria for judgment of overriding appropriateness, a group of 6 trained clinicians consisting of physicians, pharmacists, and nurses worked together. We adopted a stepwise approach recommended by the Kidney Disease: Improving Global Outcomes guidelines of 2010 to improve drug dosing [11, 12]. The stepwise approach facilitates inclusion of multiple considerations to achieve the desired goal in a timely manner for each drug. Similarly, we considered multiple data in a stepwise manner. First, we examined the values and trend of eGFR or estimated CrCL for the last 6 months to determine if there was a steadily moderate or severe decrease in renal function. Second, we reviewed electronic medical records of demographics, history of disease, physician notes, and laboratory results to obtain evidence to support the override reason(s) entered by a provider, i.e., to determine whether a patient has tolerated the drug in the past, whether new evidence supports the type of therapy, or whether a consultant was approached. Third, we reviewed the medication history, monitored drug responses, and revised the regimen after assessing whether substitute drugs were used before (Figure 2). The initial criteria were modified iteratively until a consensus was reached, with over 90% agreement for examination of a sample of 50 randomly selected overrides.

### Sampling and chart review

Among the override prescriptions, we limited the samples to providers with more than 20 alerts because of the opportunity to override for 3 years. We randomly sampled 300 warnings stratified by the prescribing provider’s override rate; 200 warnings from the providers were categorized in the top 25 warnings with high-frequency overrides and 100 warnings were taken from the remaining providers. Using the override appropriateness criteria, a researcher (I.C.) reviewed the LMR and abstracted relevant information for 300 overrides from the date the alert was triggered. Subsequently, each case was reviewed by an attending physician (K.C.N.). The final kappa value of inter-rater reliability was excellent (0.93). Disagreements were resolved by discussion with a third reviewer (D.W.B.). The reviewers were aware that they could be reviewing frequent overriders, but they did not know the override rates for individual providers.

### Outcomes

Our primary outcome was the rate of override appropriateness, which indicates how many overrides could be regarded as clinically justifiable. This outcome was calculated as the ratio of the number of appropriate overrides to the total number of overrides. Our secondary outcomes were the rate of appropriate overrides by provider type, prescription type, drug type, and override reason(s). Alert override rate and adherence rate were calculated based on total alerts.

### Data collection and analysis

We obtained renal alert override rates and the coded reasons for overrides cited by providers at the time of prescription from outpatient clinics and ambulatory hospital-based practices over a period of 3 years, from January 1, 2009 to December 31, 2011. All provider types, including staff physicians, house staff, and nurse practitioners, were considered non-physician providers with prescribing authority.

Descriptive statistics were used to summarize the rates of alert overrides and appropriateness. We compared the appropriateness of overrides between sampling units and provider types by drug and override reason. In the detailed review of the 300 cases, we excluded redundant cases (*n* = 5), cases in which target medications were cancelled later (*n* = 4), and cases for which we could not retrieve the electronic medical records (*n* = 2). Thus, 194 cases from the 200 samples and 95 from the 100 samples were analyzed. Data are presented as numbers with percentages, and *P* values were calculated using the Chi-square test. We used SAS 9.3 software (SAS Institute, Cary, NC, USA) for data analyses.

## Results

### Incidence of renal alerts and overrides

**Figure 3:**
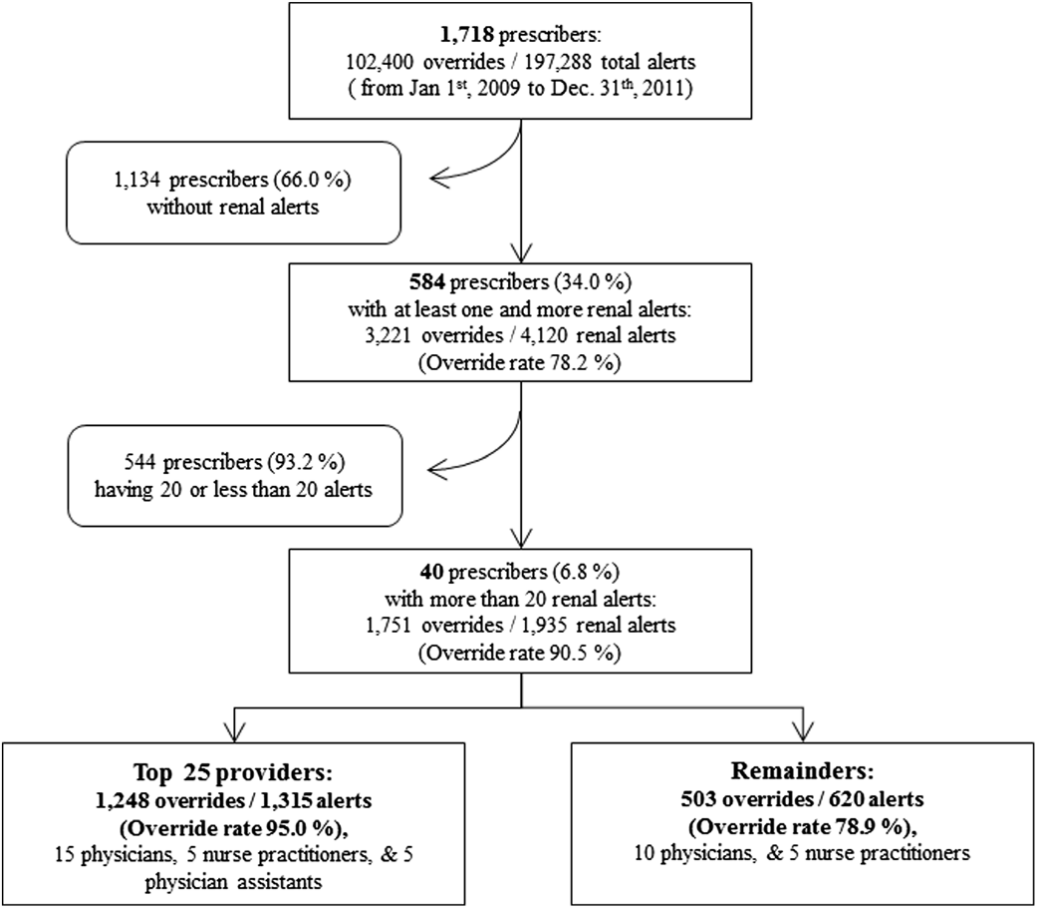
**Breakdown of alert and override frequency by prescribers.**

For the 3-year period, total alerts triggered were 197,288, by 1,718 prescribers along with all types of medication in the CDS system, which included renal alerts. Among the alerts, 4,120 were renal alerts and were triggered by 584 prescribers (34% of all prescribers), and 3,221 (78.2%) were overridden (Figure 3). Among the prescribers, 544 (93.2%) had triggered ≤20 alerts. This group had an average override rate of 70%. The remaining 40 providers (6.8%) had triggered >20 alerts and had triggered almost half of the renal alerts, with a 90.5% override rate. On classifying the providers into the top 25 overriders (high-frequency overriders) and the remaining overriders (low-frequency overriders), the override rates were 95.0% and 78.9%, respectively.

### Override appropriateness

Based on the override appropriateness criteria, the electronic medical records of 289 override samples by 35 providers were examined. Of 289 overrides, 85 (29.4%) prescriptions were considered appropriate (Table 1). Low-frequency overriders showed a slightly higher appropriateness rating than high-frequency overriders. Most of the appropriate overrides were for prescriptions given for a temporal drop or fluctuation in the CrCL value below a specific drug’s threshold (e.g., 50 mL/min for metformin and 30 mL/min for hydrochlorothiazide) or reference CrCL value, which was on the borderline. In some cases, the CrCL values were missing from the LMR; therefore, referenced eGFR values were used instead of CrCL values. For 11 overrides, providers had tried to use substitute drugs or initiated drugs with minimum doses.

**Table 1:** **Frequency of overrides and appropriate overrides by provider group**

	Frequency (%)		
Prescription provider group	Overridden alerts	Appropriate overrides	Inappropriate overrides
High frequency over-riders (*n* = 25)	194 (100.0)	55 (28.4)	139 (71.6)
Low frequency over-riders (*n* = 10)	95 (100.0)	30 (31.6)	65 (68.4)
Total	289 (100.0)	85 (29.4)	204 (70.6)

We compared the proportion of appropriate overrides by provider type (Table 2) and found that 5 prescribers were physician assistants who entered 39 medication prescriptions in the top 25 samples. We excluded them because we were not sure whether the physician assistants’ supervising physician(s) saw the alerts directly and responded to them. A physician assistant might ask the physician whether there was an alert and how to respond it. Considering the relationship between the physician and the assistant regarding entering prescriptions into system, it is not plausible for a physician to read the information on the alert by himself/herself. We further looked into 250 prescriptions entered by 21 physicians and 9 nurse practitioners to compare the rates. Staff physicians (*n* = 20) and house staff (*n* = 1) showed a higher proportion of appropriate overrides (33%) than nurse practitioners (22%). On categorizing the overrides by drug, glyburide showed the highest number of appropriate overrides, followed by hydrochlorothiazide, metformin, nitrofurantoin, and the others. On categorizing by prescription type, renewal medications were more likely to be overridden and showed higher appropriate overrides than new medications.

**Table 2:** **Rates of appropriate override by provider type, drug type, prescription type, and override reason(s)**

	No. of appropriate overrides/no. of overridden alerts (%)		
	Physicians (***n*** = 21)	Nurse practitioners (***n*** = 9)	Total
Drug type			
Metformin	25/79 (31.6)	11/37 (29.7)	36/116 (31.0)
HCTZ	18/50 (36.0)	5/16 (31.3)	23/66 (34.8)
Glyburide	12/26 (46.2)	1/8 (12.5)	13/34 (38.2)
Nitrofurantoin	2/12 (16.7)	0/14 (0.0)	2/26 (7.7)
Others^†^	0/6 (0.0)	0/14 (0.0)	0/20 (0.0)
Prescription types			
New	2/23 (8.7)	0/21 (0.0)	2/43 (4.7)
Renew	55/150 (36.7)	17/56 (30.4)	72/201 (35.8)
Override reasons			
Patient has tolerated this drug in the past	43/123 (35.0)	15/57 (26.3)	58/180 (32.2)
New evidence supports therapy of this type	8/18 (44.4)	0/2 (0.0)	8/20 (40.0)
Advice from a consultant	1/1 (100.0)	0/2 (0.0)	1/3 (33.3)
Other^‡^	5/31 (16.1)	2/16 (12.5)	7/47 (14.9)
Total	57/173 (32.9)	17/77 (22.1)	74/250 (29.6)

HCTZ, hydrochlorothiazide. Others^†^ includes acetylsalicylic acid, and combination of aspirin and oxycodone. Other^‡^ indicates a category.

Regarding the reasons for override, most providers chose the “patient has tolerated this drug in the past” option to reason the override alert. As such, the trend of CrCL or eGFR values for the last 6 months should was stable over the moderate category. However, sometimes, it was difficult to find such evidence in the chart review, which was similar for cases of both appropriate and inappropriate overrides. For example, the 8 prescriptions in which physicians selected the “new evidence supports therapy of this type” as an override reason were judged as appropriate because the CrCL values were maintained around the threshold or the physicians had used a substitute drug first or minimum-dose approach at initiation. For the “other” category option, no additional text was entered.

On comparing provider groups by drug, physicians who were low-frequency overriders showed significantly higher override appropriateness than physicians who were high-frequency overriders for metformin (Table 3). For hydrochlorothiazide, the physicians of the high-frequency overriders were more accurate in their decision than the low-frequency overriders, although this difference was not significant. Glyburide showed the highest appropriateness rate of 80%.

**Table 3:** **Comparison of appropriate override rates between provider groups by drug**

Provider drug	No. of appropriate overrides/no. of overridden alerts (%)					
	Physicians (***n*** = 21)			Nurse practitioners (***n*** = 9)		
	High frequency overriders	Low frequency overriders	***χ*** ^***2***^(***P***) ^†^	High frequency overriders	Low frequency overriders	***χ*** ^***2***^(***P***) ^†^
Metformin	11/53 (20.8)	14/26 (53.9)	8.83 (0.0046)	7/20 (35.0)	4/17 (23.5)	0.58 (0.4951)
HCTZ	14/34 (41.2)	4/16 (25.0)	1.24 (0.3510)	2/7 (28.6)	3/9 (33.3)	0.04 (1.000)
Glyburide	8/21 (38.1)	4/5 (80.0)	2.85 (0.1478)	1/5 (20.0)	0/3 (0.0)	0.69 (1.000)
Nitrofurantoin	1/6 (16.7)	1/6 (16.7)	0.0 (1.000)	0/7 (0.0)	0/7 (0.0)	0.0 (1.000)
Other^†^	0/2 (0.0)	0/4 (0.0)	0.0 (1.000)	0/12 (0.0)	0/2 (0.0)	0.0 (1.000)
Total	34/116 (29.3)	23/57 (40.4)	2.11 (0.1465)	10/39 (25.6)	7/38 (18.4)	0.58 (0.5843)

HCTZ, hydrochlorothiazide. Other^†^ includes acetylsalicylic acid, and combination of aspirin and oxycodone. ^†^Indicates a Fisher’s exact test.

Evaluation of the proportion of appropriateness by prescription types showed no significant differences (Table 4). Renewal medications of physicians were more frequently overridden and appropriate than the new medications. On comparison of provider groups, physicians who were low-frequency overriders had higher appropriateness than those who were high-frequency overriders. Among nurse practitioners, no appropriate override was noted for a new medication.

**Table 4:** **Comparison of appropriate override rates between provider groups by prescription type**

Provider prescription type	No. of appropriate overrides/no. of overridden alerts (%)					
	Physicians (***n*** = 21)			Nurse practitioners (***n*** = 9)		
	High frequency overriders	Low frequency overriders	***χ*** ^***2***^(***P***) ^†^	High frequency overriders	Low frequency overriders	***χ*** ^***2***^(***P***) ^†^
New	1/16 (6.3)	1/7 (14.3)	0.40 (0.5257)	0/12 (0.0)	0/9 (0.0)	0.0
Renew	33/100 (33.0)	22/50 (44.0)	1.74 (0.1875)	10/27 (37.0)	7/29 (24.1)	1.10 (02942)

^†^Indicates a Fisher’s exact test.

## Discussion

In our study, one of every 3 renal alerts was triggered by high-frequency overriders who also overrode alerts more often than those who received fewer alerts. Overall, approximately 30% of the overrides were appropriate for both physicians and nurse practitioners. Only 3 drugs—metformin, hydrochlorothiazide, and glyburide—accounted for the majority of overridden prescriptions.

Our findings show a slightly different perspective to the noncompliance problem of renal-dosing guidelines and promote a CDS approach to reduce kidney-related drug-prescription errors. Taking into account the previous works, reporting the eGFR with other measures of kidney function has been no effect on improving the compliance, and a wide availability of drug-dosing guidelines was not sufficient [13]. As current studies have repetitively reported, the frequency of excess dosing of kidney disease-related drugs in older patients with CKD in an ambulatory setting is still high [5, 13]. Such noncompliance problems are similar to those seen in hospitalized patients and residents of long-term care facilities [14, 15]. A CDS system approach was regarded as a new tool to provide the necessary framework to reduce inappropriate drug prescription. However, the CDS system alone might not be sufficient. In a previous study on the effects of a CDS system in patients with renal insufficiency, Chertow et al. found that despite the overall improved appropriateness of dosing, 49% of medication orders were still inappropriate in the intervention group [8].

Studies that reported successful effects of a CDS system for patients with renal insufficiency showed similar limitations. One study [16] revealed an improvement in many aspects of prescription including improved frequency of administration and lower rates of orders for drugs that should be avoided. However, the system did not improve the rate at which physicians ordered appropriate doses for residents with renal insufficiency. On comparison by alert type, appropriate orders ranged from 41–75% in the CDS intervention group; inappropriate orders still accounted for 25–49% of the total orders. Another study [17] reported that a CDS approach decreased the likelihood of a patient receiving at least one dose of a contraindicated drug, but the patients still had a 47% chance of being prescribed a contraindicated drug. Regarding the limitations of CDS, some physicians may have been reluctant to reduce the drug dosages, and others may have simply disregarded the advice, favoring their own established practice patterns. In the present study, we could depict really they are and be relatively small number as high overriders. If we could approach and intervene them, we could improve the noncompliance problemoverall. In other words, physician-level targeted interventions might be helpful to improve high override rates if the physicians can be assured of the importance of the warnings.

With regard to override appropriateness, little data on renal suggestion alerts are available. On comparing the override appropriateness of other alert types, the rate reported in the present study (29.4%) was lower than that of override appropriateness of drug interactions in our previous work (63%) [18] and of drug interaction and drug allergy in another previous study (63.5%) [1]. These differences should be considered owing to the relatively clear rules about renal insufficiency by drugs. McCoy et al. [19] presented a framework using the matrix of alertness displayed and provider response. They defined the rate of inappropriateness as the ratio of the number of inappropriate responses to the total number of alerts. However, in the current study, we focused on providers’ appropriate override rather than appropriate alerts. The rate of inappropriateness was calculated as the ratio of the number of unjustifiable overrides to the number of total overrides. The unjustifiable overrides were those that were judged as clinically unacceptable by the override appropriateness criteria. Based on the rate of appropriate overrides, we can expect that a decrease of approximately 30% in the override rates would indicate reasonable improvement in providers’ decision-making. For the borderline cases, it would be helpful to inform providers about the variations and trends in CrCL or eGFR. Regarding the subgroup analysis by provider type, physicians’ rates of appropriate overrides were higher than those of nurse practitioners with regard to drug type, prescription type, and override reason(s). This implies that more support is needed for nurse practitioners. In addition, more awareness about the appropriate use of metformin, hydrochlorothiazide, and glyburide is needed. As Huang et al. [20] reported, CKD was common in older adults prescribed metformin for type 2 diabetes, and this is a growing concern for potentially inappropriate medication use.

Our findings were consistent with those of previous studies, which reported override rates ranging from 40% to >80% with CDS for nephrotoxic or renally cleared medications in various settings [7, 17, 21]. The override rate in the present study (78.2%) was relatively higher than that reported previously in studies conducted in inpatient settings and the emergency department [14, 17]. One study showed physicians overrode 59.4% of alerts, recommending that a drug be avoided [14]. Another study reported a 43% overdosing rate after implementation of CDS alerts [17]. McCoy et al. [22] observed that in 78.1% of the cases, providers initially deferred interruptive alerts indicating contraindicated or high-toxicity medications. Using the same medication dosing system as in the present study, Chertow et al. demonstrated that inappropriate prescription rates decreased to 33% and 41% by dose and frequency, respectively, for inpatients with renal insufficiency [8]. Our current override rate, which is higher, suggests that the number of alerts that our providers experienced has increased, which could lead to alert fatigue.

There are three possible reasons for this high override rate. First, the override rate may differ depending on the clinical setting. Outpatients might be tend to be older and have chronic diseases such as hypertension or/and diabetes compared to inpatients of acute care. We noted that the major drugs overridden were hypertension and diabetes medications, which accounted for 86% of the overridden drugs. We also found that a small number of drugs were overridden repetitively over patients; this finding was limited to outpatients, and metformin or hydrochlorothiazide was the most commonly overridden drugs in other settings. Previous studies have reported various drug lists involved in overriding alerts. To our knowledge, the current study is the first to report on the use of renal suggestions in an outpatient setting. Therefore, we need more studies to elaborate on this difference in varied settings. Second, it is possible that high-frequency overriders substantially contributed to the increase override rate. Third, a crude increase in the number of alerts due to addition of new alert types over time on the computerized physician order entry systems or known drugs requiring alerting could increase the total number of alerts. Over the years, the alerts displayed have been undergoing revision, and more low-yield warnings may have been added [23]. Considering renal suggestions only, the alert thresholds for outpatients were adjusted higher than that for mild renal insufficiency (estimated CrCL, 50–80 ml/min). In addition, when we examined whether the top 25 high overriders overlapped with the high-frequency overriders of other types of alerts, we found that more than two-thirds overlapped with the high-frequency overriders of geriatrics, drug interactions, and drug-allergy types. This implies that the effect of a crude increase in the number of alerts is considerable.

Regarding the reasons cited by providers, we found the coded reason(s) were not used accurately by providers and did not reflect any clinical context. This finding was similar to that of our previous work on drug interaction, wherein we found that only one-third of the providers overrode according to override reason(s) and sometimes, we had no evidence to confirm the reason(s) [18]. The list of coded reason(s) might not be suitable for delivering clinical context or providers simply believe that choosing an accurate reason was not important. Our findings suggests that organizations should consider periodically reviewing the reasons selected for overrides—if providers know that someone is evaluating this information, they may be more likely to provide accurate information. In order to account for these override reason(s) while extrapolating override appropriateness and while setting up a management goal, collecting data correctly is important.

Our study has several limitations. First, it was undertaken within a single academic health care center using one outpatient-prescribing system, and the results may not be generalizable to other prescribing systems. Nevertheless, the findings have important implications for enhancing medication safety and identifying an approach to decrease high override rates. Second, we did not evaluate the appropriateness by patient and could not follow up patients’ results of inappropriate overrides. However, inappropriate overrides reflect, to some extent, medication errors that accompany kidney disease, which can affect every organ system and every aspect of drug disposition.

## Conclusions

The outpatient setting includes the elderly and many patients with chronic diseases, i.e., groups that are especially vulnerable to inappropriate medication dosing and drugs. Overall, 3 of 10 overrides were regarded as clinically justifiable. A small number of providers accounted for a large proportion of overrides, and they nearly always overrode inappropriately. These data suggest that a provider-level intervention might successfully improve medication safety in this group of patients.

## Authors’ information

IC is affiliated with the Division of General Internal Medicine, Brigham and Women’s Hospital, Boston, Massachusetts, USA, and the Department of Nursing, Inha University, Incheon, South Korea.
